# Investigating the Role of the NLRP3 Inflammasome Pathway in Acute Intestinal Inflammation: Use of THP-1 Knockout Cell Lines in an Advanced Triple Culture Model

**DOI:** 10.3389/fimmu.2022.898039

**Published:** 2022-07-13

**Authors:** Mathias Busch, Haribaskar Ramachandran, Tina Wahle, Andrea Rossi, Roel P. F. Schins

**Affiliations:** Institut für Umweltmedizinische Forschung (IUF) – Leibniz-Research Institute for Environmental Medicine, Duesseldorf, Germany

**Keywords:** caco-2, HT29-MTX-E12, gastrointestinal tract, IBD, barrier, inflammatory disease, mucus, macrophages

## Abstract

The NLRP3 inflammasome plays an important role in intestinal homeostasis as well as inflammation. However, *in vivo* studies investigating the role of the NLRP3 inflammasome in inflammatory bowel disease (IBD) report contrasting results, leaving it unclear if the NLRP3 inflammasome augments or attenuates intestinal inflammation. To investigate the role of the NLRP3/caspase-1 pathway in a model of acute intestinal inflammation, we modified a previously established *in vitro* triple culture model of the healthy and inflamed intestine (Caco-2/HT29-MTX-E12/THP-1). Using THP-1 knockout cell lines, we analyzed how the NLRP3 inflammasome and its downstream enzyme caspase-1 (CASP1) affect inflammatory parameters including barrier integrity and cytotoxicity, as well as gene expression and secretion of pro-inflammatory cytokines and mucus. Furthermore, we investigated differences in inflammation-mediated cytotoxicity towards enterocyte-like (Caco-2) or goblet-like (HT29-MTX-E12) epithelial cells. As a complementary approach, inflammation-related cytotoxicity and gene expression of cytokines was analyzed in intestinal tissue explants from wildtype (WT) and *Nlrp3^-/-^* mice. Induction of intestinal inflammation impaired the barrier, caused cytotoxicity, and altered gene expression of pro-inflammatory cytokines and mucins *in vitro*, while the knockout of *NLRP3* and *CASP1* in THP 1 cells led to attenuation of these inflammatory parameters. The knockout of *CASP1* tended to show a slightly stronger attenuating effect compared to the *NLRP3* knockout model. We also found that the inflammation-mediated death of goblet-like cells is NLRP3/caspase-1 dependent. Furthermore, inflammation-related cytotoxicity and upregulation of pro-inflammatory cytokines was present in ileal tissue explants from WT, but not *Nlrp3^-/-^* mice. The here presented observations indicate a pro-inflammatory and adverse role of the NLRP3 inflammasome in macrophages during acute intestinal inflammation.

## Introduction

Inflammatory bowel disease (IBD), namely Crohn’s disease (CD) or ulcerative colitis (UC) are characterized by chronic, relapsing inflammation of the gastrointestinal tract (1). IBD is thought to originate from an exaggerated immune response towards constituents of the mucosal microbiome, but the precise etiology is still not known (2, 3). As a central activator of the innate immune system, the NOD-, LRR- and pyrin domain-containing protein 3 (NLRP3) inflammasome is a crucial regulator of intestinal homeostasis and has been implicated in the pathogenesis of IBD (4, 5). After activation *via* pathogen-associated molecular patterns (PAMPs) or damage-associated molecular patterns (DAMPs), the NLRP3 inflammasome oligomerizes and recruits procaspase-1, which leads to the formation of the active caspase-1 (CASP1) (6). CASP1 cleaves the inactive pro-IL-1β, which results in the secretion of mature IL-1β (7). As an early pro-inflammatory cytokine, IL-1β induces subsequent inflammatory cascades (8), including the activation of the transcription factor NF-κB (9). In turn, NF-κB regulates secondary inflammatory cytokines like IL-6 and IL-8 (10, 11).

However, the exact role of the NLRP3 inflammasome in IBD is not clear (12), as different studies report conflicting results: genetic polymorphisms decreasing *NLRP3* expression in patients were linked to higher susceptibility towards CD (13), while *Nlrp3* knockout mice developed less severe DSS-induced colitis compared to the wild type (WT) (14). The latter effect could be reversed by cohousing *Nlrp3^-/-^* mice with WT mice (15), which implies a crucial role of the microbiome in IBD susceptibility. Other studies reported increased colitis symptoms in *Nlrp3^-/-^* mice when compared to WT mice (16, 17).

The gastrointestinal epithelium is covered in mucus, which plays an important protective role in intestinal homeostasis. Mucus primarily consists of different mucins, highly glycosylated proteins mainly produced and secreted by goblet cells, but also in parts by enterocytes. Mucins can be divided into two classes: secreted mucins and membrane-associated mucins. Secreted mucins, such as MUC2 and MUC5AC, and also transmembrane mucins, such as MUC1, MUC13, and MUC20, have been shown to be altered or aberrantly expressed in patients suffering from IBD (18–23). This indicates a major role of mucus not only during homeostasis, but also during intestinal inflammation.

We have previously developed an *in vitro* triple culture model of the healthy and inflamed intestine, comprised of the human cell lines Caco-2, HT29-MTX-E12, and THP-1. This system is capable of replicating several hallmarks of intestinal inflammation, including impairment of barrier integrity, cytotoxicity, an increase of pro-inflammatory cytokine secretion, reduction of acidic mucus secretion, and DNA damage in epithelial cells after prolonged inflammation (24, 25). In these previous studies, this model was applied in toxicological research investigating particle effects in the context of inflammation.

The aim of the present study was to investigate the role of the NLRP3 inflammasome and its downstream enzyme CASP1 in macrophages during initial acute intestinal inflammation by using the *in vitro* triple culture model with different THP-1 cell lines (WT, *CASP1^-/-^* and *NLRP3^-/-^*). As a complementary approach, we studied short-term inflammation-related cytotoxicity and gene expression of pro-inflammatory cytokines in intestinal tissue explants from WT and *Nlrp3^-/-^* mice.

## Materials and Methods

### Materials

The cell culture media (DMEM, MEM, RPMI 1640), 2-mercaptoethanol (ME), horse serum, sodium pyruvate, and phosphate-buffered saline (PBS) were purchased from Thermo Fisher Scientific. Fetal calf serum (FCS) for Caco-2 and HT29-MTX-E12, non-essential amino acids (NEAA), L-glutamine, D-glucose, penicillin/streptomycin (P/S), trypsin, phorbol 12-myristate 13-acetate (PMA), interferon-gamma (IFN-γ), lipopolysaccharides (LPS), Accutase, β-nicotinamide adenine dinucleotide sodium salt (NAD), lithium L-lactate, phenazine methosulfate (PMS), iodonitrotetrazolium chloride (INT), Triton X-100, bovine serum albumin (BSA), and 50 nm amine-modified polystyrene nanobeads (PS-NH_2_) were purchased from Sigma-Aldrich/Merck. Paraformaldehyde (PFA) was ordered from Carl Roth (Germany). The DQ12 quartz sample used in this study was kindly provided by the Institute of Occupational Medicine (IOM, Edinburgh, UK).

### Cell Culture

Caco-2 (DSMZ, ACC169) cells were cultured in MEM-based cell culture medium substituted with 20 % FCS, 1 % P/S and 1 % L-glutamine. HT29-MTX-E12 (ECACC, 12040401) cells were cultured in DMEM-based cell culture medium substituted with 10 % FCS, 1 % P/S, and 1 % NEAA. Caco-2 and HT29-MTX-E12 cells were regularly split at roughly 80 % confluence and used at passages 5 – 25 for experiments. THP-1 (ATCC, TIB-202) cells were cultured in RPMI 1640-based cell culture medium (containing L-glutamine and 25 mM HEPES) substituted with 10 % FCS, 1 % P/S, 1 nM sodium pyruvate, 0.7 % D-glucose and 50 nM ME and maintained between 2*10^5^ and 8*10^5^ cells/ml. For experiments, THP-1 cells were used at passages 5 – 15.

### Generation of CASP1-Deficient and NLRP3-Deficient THP-1 Cell Lines

*CASP1* and *NLRP3* THP-1 mutant cells were generated as previously described (26). Briefly, gRNAs were designed using the CRISPR design tool CHOPCHOP (http://chopchop.cbu.uib.no/) and cloned into a modified PX458 plasmid (Addgene #48138). The resulting bicistronic vector encoded the respective gRNA, Cas9 nuclease, and a GFP selection marker. gRNAs efficiency was assessed using High-Resolution Melt Analysis (HRMA). A gRNA targeting *CASP1* exon 5 (5’-TAATGAGAGCAAGACGTGTG-3’) and *NLRP3* exon 2 (5’-GCTAATGATCGACTTCAATG-3’) were chosen for further experiments (HRMA primers, *CASP1*: Fwd 5’-CACCGTAATGAGAGCAAGACGTGTG-3’ Rev 5’-AAACCACACGTCTTGCTCTCATTAC-3’; *NLRP3*: Fwd 5’-CAGACCATGTGGATCTAGCC-3 Rev 5’-TGTTGATCGCAGCGAAGAT-3’). THP-1 cells were electroporated using a Neon transfection system (Thermofisher) according to the manufacturer’s instructions. After 48 h, cells were FACS sorted and plated as single cells into 96-well plates. Cells were duplicated into maintenance and lysis plates after a week. Clones were then lysed with proteinase K and genotyped by PCR followed by deep sequencing using a miSeq Illumina sequencer and a V2 Nano cassette.

### Investigation of the Cytokine Profile of THP-1 ko Cell Lines

THP-1 cells (WT, *CASP1^-/-^* and *NLRP3^-/-^*, at the same passage number) were seeded into T25 flasks at 6*10^5^ cells/ml, differentiated with 100 nM PMA for 24 h, detached with accutase, and seeded in 24-well plates at 120.000 cells/well. Cells were allowed to re-attach for 1.5 h and were treated with different NLRP3 inflammasome activators in RPMI-based medium containing 1% FCS: 10 µg/cm² amino-modified polystyrene nanospheres (PS-NH_2_), 200 µg/cm² crystalline silica (DQ12), 10 ng/ml LPS or a combination of 10 ng/ml LPS and 10 ng/mL IFN-γ. After 24 h, supernatants were collected and stored at -20°C until analysis *via* ELISA.

### Stable and Inflamed Triple Cultures

The set-up of the stable and inflamed triple cultures was established as described previously (24). In this context, the terms “stable” and “inflamed” relate to the physiological states of the intestine that the model aims to represent. Briefly, Caco-2 and HT29-MTX-E12 cells were seeded in transwell inserts (12-well format) and grown for 21 days to allow differentiation of Caco-2 and mucus production by HT29-MTX-E12 cells. For the stable triple culture, the inserts were transferred onto PMA-differentiated THP-1 cells seeded in 12-well plates (WT, *CASP1^-/-^* and *NLRP3^-/-^*, at the same passage number). For the inflamed triple culture, epithelial cells were primed with 10 ng/ml IFN-γ for 24 h and then transferred onto PMA-differentiated THP-1 cells, which were pre-activated with 10 ng/ml LPS/IFN-γ for 4 h. Stable and inflamed triple cultures were started in parallel and maintained for 48 h.

### Cytokine Quantification by Enzyme-Linked Immuno-Sorbent Assay

The release of IL-1β, IL-8, and TNF-α into the supernatant of THP-1 monocultures (t=24 h) or the basolateral supernatant of triple cultures (t=48 h) was analyzed using R&D systems DuoSet ELISA kits for these proinflammatory cytokines. Antibodies were diluted according to the manufacturer’s protocol and high-protein-binding 96-well plates were incubated with capture antibody in coating buffer (0.1 M NaHCO_3_, pH 8.2) overnight. After blocking with 3% BSA/PBS, 100 µl of supernatants, diluted if necessary (IL-1β: 1:5 and IL-8: 1:10), were incubated for 2 h. Detection antibody, horseradish peroxidase (1:40 in 1% BSA/PBS), and BioRad TMB Peroxidase EIA Substrate was consecutively incubated for 2 h, 0.5 h, and 5-20 min, respectively, before stopping the reaction with 50 µl 1 M H_2_SO_4_. Absorbance was measured at 450 nm and 540 nm. The standard curve was plotted as a four-parameter log fit.

### Measurement of Transepithelial Electric Resistance

Barrier integrity during triple cultures was measured as TEER using a voltohmmeter (EVOM, World Precision Instruments) with a chopstick electrode (STX2). Before measurements, the electrode was sterilized in 70 % ethanol and washed with PBS and MEM. TEER measurements were performed at t = 0,4,20,24,44,48 after the start of the triple cultures and were corrected for blank value and transwell filter area (0.9 cm²).

### LDH Assay

To investigate cytotoxicity in the epithelial cells after 48 h of triple culture, 50 µl of apical supernatant was transferred onto 96-well plates and 150 µl of reaction mix containing 50 µl Tris buffer (200 mM, pH 8), 50 µl Li-Lactate solution (50 mM), 46 µl NAD^+^ solution (5 mM), 2 µl INT solution (65 mM) and 2 µl PMS solution (29 mM) was added per well. After 5 min incubation, the reaction was stopped with 50 µl 1 M H_2_SO_4,_ and absorbance was measured at 490 nm and 680 nm.

### Analysis of Cell-Specific Cytotoxicity During Inflammation

To analyze differences in cytotoxicity between Caco-2 and HT29-MTX-E12 cells during inflammation, 10^4^ Caco-2 or HT29-MTX-E12 cells per well were seeded in 96-well plates. After 48 h of growth, cells were treated with basolateral supernatants [t=48h; relative concentrations of 0.25, 0.5 and 1; diluted with RPMI-based medium (e.g. concentration of 0.25: 25 % supernatant in medium; 1: undiluted supernatant)]. Treatment with 0.5% Triton-X 100 for 15 min served as positive control, supernatant at the relative concentrations without cells served as blanks. After 48 h incubation, the LDH assay was performed as described above. Cytotoxicity was calculated as follows: $\%\;Cytotoxicity=\frac{sample-blank}{pos.\;\;control-blank}$ .

### Immunofluorescent MUC5AC Staining

To further investigate the cell-specificity of inflammation-derived cytotoxicity, the epithelial cell layer was stained for MUC5AC, a mucin produced by the goblet-like HT29-MTX-E12 cell line, but not Caco-2 (27). After 48 h of triple culture, the filters were fixed in 4 % paraformaldehyde, permeabilized in 0.1 % Triton-X 100/PBS, and blocked with 3 % BSA/PBS. Filters were incubated with MUC5AC primary antibody (mouse; 2 µg/ml) in 1 % BSA/PBS for 1 h at RT and with Alexa 488 goat-anti-mouse antibody (1:300) and Hoechst 33342 (0.5 µg/ml) for 30 min at 37°C. Filters were placed on microscopy slides, mounted with Prolong Gold Antifade, and sealed with a cover slip. Microscopic evaluation was performed using a Zeiss Axio Imager.M2 at 100x magnification. For each condition, 20 images were taken and MUC5AC coverage was quantified as fluorescent area [%], using a macro in ImageJ version 1.53c (see **Supplementary Material**).

### Gene Expression Analysis

Following 48 h of triple culture, gene expression was investigated in the epithelial cell layer and THP-1 cells separately. RNA was isolated using the Roche High Pure RNA Isolation kit. Filters containing epithelial cells were cut out of the inserts using a scalpel and placed in 200 µl ice-cold PBS. THP-1 cells were collected from the well bottom using a cell scraper in 200 µl PBS. 400 µl lysis buffer was added to each sample and vortexed for 15 s. Subsequently, the RNA isolation kit was used according to the manufacturer’s instructions. RNA quantification, DNase I digestion with the Amplification Grade DNase I Kit (Sigma-Aldrich), reverse transcription with the iScript cDNA Synthesis Kit (Bio-Rad), and qRT-PCR were performed as described previously (25). The expression of *IL-1β*, *IL-8*, and *TNF-α* was investigated in both epithelial and THP-1 cells and the expression of *MUC1*, *MUC2*, *MUC5AC*, *MUC13*, and *MUC20* was analyzed in epithelial cells only. *β-actin* was used as reference gene. Primers are listed in **Supplementary Table 1**. Changes in gene expression dependent on inflammation status, or dependent on THP-1 cell line were calculated using the ΔΔC_T_ method (28).

### Cultivation and Treatment of Ileal Tissue Explants

Ileal tissue was collected from healthy mice (C57BL6/J) under the study approval by the Landesamt für Natur, Umwelt und Verbraucherschutz (LANUV, Northrhine Westphalia, Germany, reference number 81-02.05.50.17.018). WT and *Nlrp3^-/-^* mice, pairs of female siblings from heterozygous parents, were sacrificed by cervical dislocation, transferred to a semi-sterile lab bench, and dissected. The ileum was isolated, rinsed with HBSS^+/+^ supplemented with 1% P/S, cut open length-wise and cut into 12 equally sized pieces (~2 mm²). Tissues were separately placed in the wells of a 24-well plate containing 1 ml HBSS^+/+^ supplemented with 1% P/S and the 24-well plate was transferred to a sterile lab bench. Using sterile forceps, the explants were transferred to a new 24-well plate containing 1 ml explant medium (DMEM, high glucose, supplemented with 10% horse serum, 1% P/S, 1 µM hydrocortisone, 14.3 nM glucagon, 1 nM 3,3’,5-triiodo-L-thyronine, 200 µM ascorbate-2-phosphate, 20 µM linoleic acid and 10 nM β-estradiol). This procedure and medium composition were adapted from Bareiss et al. (29). 6 of the 12 explants were immediately treated with 1 µg/ml LPS and murine IFN-γ and incubated at 37 °C and 5% CO_2_ for 6 h. Pilot studies indicated that the viability of these tissue explants decreased significantly when cultivated for longer than 6 h. After 1, 3, and 6 h, 100 µl of supernatant was collected, stored at -20°C, and replaced with 100 µl of fresh medium. The LDH assay was performed with 50 µl of supernatants as described above. 50 µl of medium without tissue served as negative control. After 6 h, tissue explants were snap-frozen in liquid nitrogen and stored at -80°C until further analysis. RNA was isolated from the intestinal tissue explants using the Roche High Pure RNA tissue kit according to the manufacturer’s instructions. Reverse transcription and qRT-PCR was performed as described above. The expression of *Il-1β*, *Il-6*, *Kc*, *Mip-2* and *Tnf-α* was analyzed using *β-actin* as reference gene and compared to the untreated control of the respective genotype. Primers are listed in **Supplementary Table 2**.

### Statistical Analysis

Statistical analysis and illustration of results were performed using GraphPad Prism 8.3. Unless stated otherwise, experiments were performed in three independent runs with two biological replicates. Statistical analysis of triple culture experiments in stable and inflamed state was performed with two-way ANOVA and Tukey’s test. A p-value of <0.05 was considered statistically significant.

## Results

First, we confirmed the successful knockout of *CASP1* and *NLRP3* in THP-1 cells by quantifying the release of pro-inflammatory cytokines after treatment with different NLRP3 activators. Next, the role of CASP1 and the NLRP3 inflammasome during intestinal inflammation was investigated in an advanced *in vitro* triple culture model by analyzing inflammation parameters including barrier integrity, cytotoxicity, gene expression, and secretion of cytokines and mucins. Furthermore, we investigated differences in susceptibility of enterocyte- and goblet-like cell types towards inflammation-derived cytotoxicity and investigated inflammation-related cytotoxicity and gene expression in intestinal tissue explants from WT and *Nlrp3^-/-^* mice.

### Reaction of THP-1 Knockout Cell Lines to Inflammatory Stimuli

**Figure 1:**
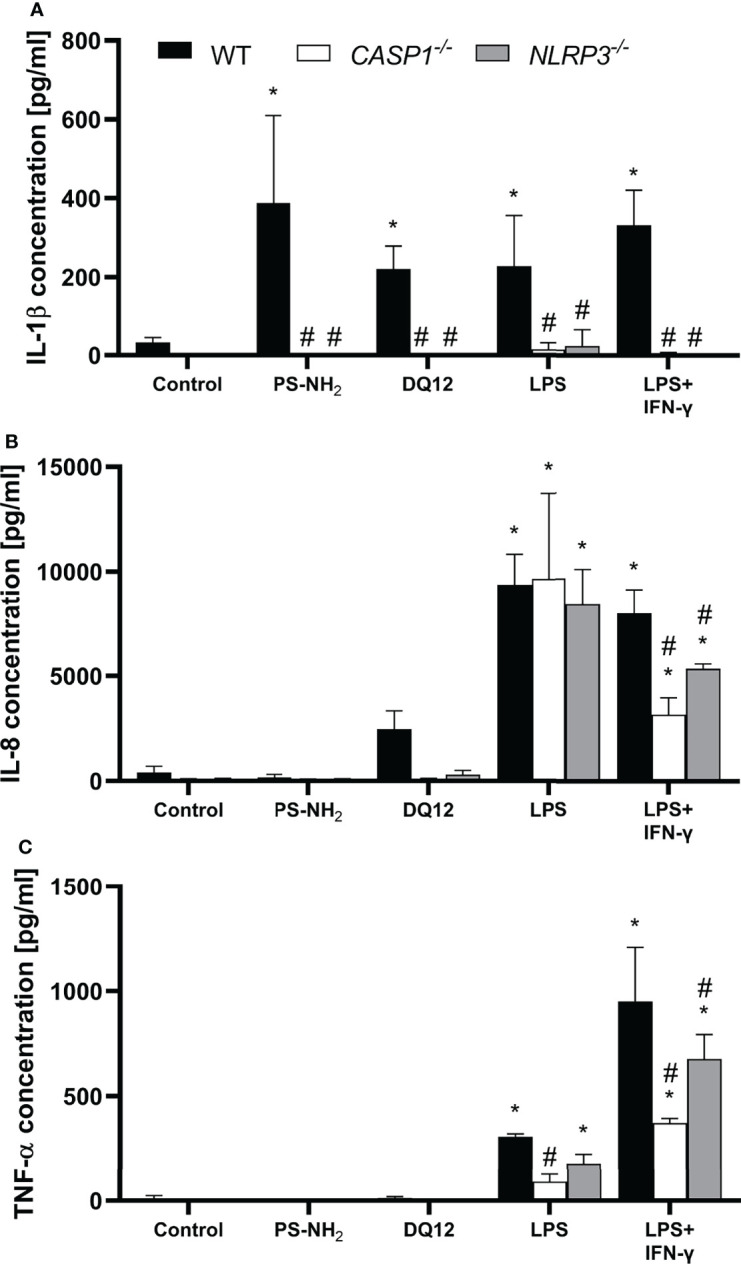
Secretion of **(A)** IL-1β, **(B)** IL-8 and **(C)** TNF-α into the supernatant by differentiated THP-1 cells (wild type, *CASP1^-/-^* or *NLRP3^-/-^*) after 24 h incubation with 10 µg/cm² amine-modified polystyrene nanobeads (PS-NH_2_), 200 µg/cm² crystalline silica (DQ12), LPS (10 ng/ml) or LSP/IFN-γ (both 10 ng/ml). Mean ± SD of N = 3; *p < 0.05, compared to the untreated control; ^#^p < 0.05, compared to the WT of the same treatment group.

To investigate the reaction of the *CASP1^-/-^* and *NLRP3^-/-^* cells towards pro-inflammatory stimuli, monocultures were treated with the different NLRP3 activators PS-NH_2_ (30), DQ12 quartz (31), LPS (32) and the combination of LPS and IFN-γ, the activators used to induce the inflamed state in the triple cultures (33). The pro-inflammatory cytokines IL-1β, IL-8, and TNF-α were analyzed after 24 h *via* ELISA (**Figure 1**).

Treatment with all NLRP3 activators induced a significantly increased secretion of IL-1β by the WT cells, which was absent in both knockout cell lines (**Figure 1A**). Treatment with LPS alone induced a similarly strong increase in IL-8 secretion in all three cell lines, while increase in TNF-α secretion was statistically significant only in WT and *NLRP3^-/-^* THP-1 cells. Treatment with LPS in combination with IFN-γ resulted in a significantly increased secretion of IL-8 and TNF-α in all three cell lines compared to the respective control, but was significantly decreased in both knockout cell lines compared to the WT. The decrease was more prominent in the *CASP1^-/-^* THP-1 cells.

### Transepithelial Electrical Resistance During Triple Culture

**Figure 2:**
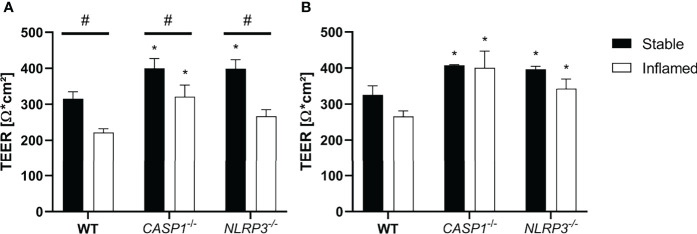
TEER at **(A)** t=24 h and **(B)** t=48 h of stable (full bars) and inflamed (open bars) triple-cultures with WT, *CASP1^-/-^* or *NLRP3^-/-^* THP-1 cells. Mean ± SD of N = 3; *p < 0.05, compared to the respective WT control; ^#^p < 0.05 compared to the respective stable culture.

Next, intestinal triple cultures were established with either WT, *CASP1^-/-^* or *NLRP3^-/-^* THP-1 cells, in both stable or inflamed state. As a primary indicator of barrier integrity, TEER was measured after 24 and 48 h (**Figure 2**).

After 24 h, TEER in inflamed triple cultures was significantly decreased in all three models, compared to the respective stable cultures (**Figure 2A**). Compared to the WT cultures, TEER of *CASP1^-/-^* cultures was significantly increased in both states, while TEER of *NLRP3^-/-^* cultures was significantly increased only in stable state. After 48 h, the differences in TEER between stable and inflamed cultures of the same model were no longer statistically significant, but TEER of stable and inflamed *CASP1^-/-^* and *NLRP3^-/-^* cultures was significantly increased compared to the respective WT cultures (**Figure 2B**). A more detailed depiction of TEER development over 48 h is given in **Supplementary Figure 1**.

### Cytotoxicity After 48 h of Triple Culture

**Figure 3:**
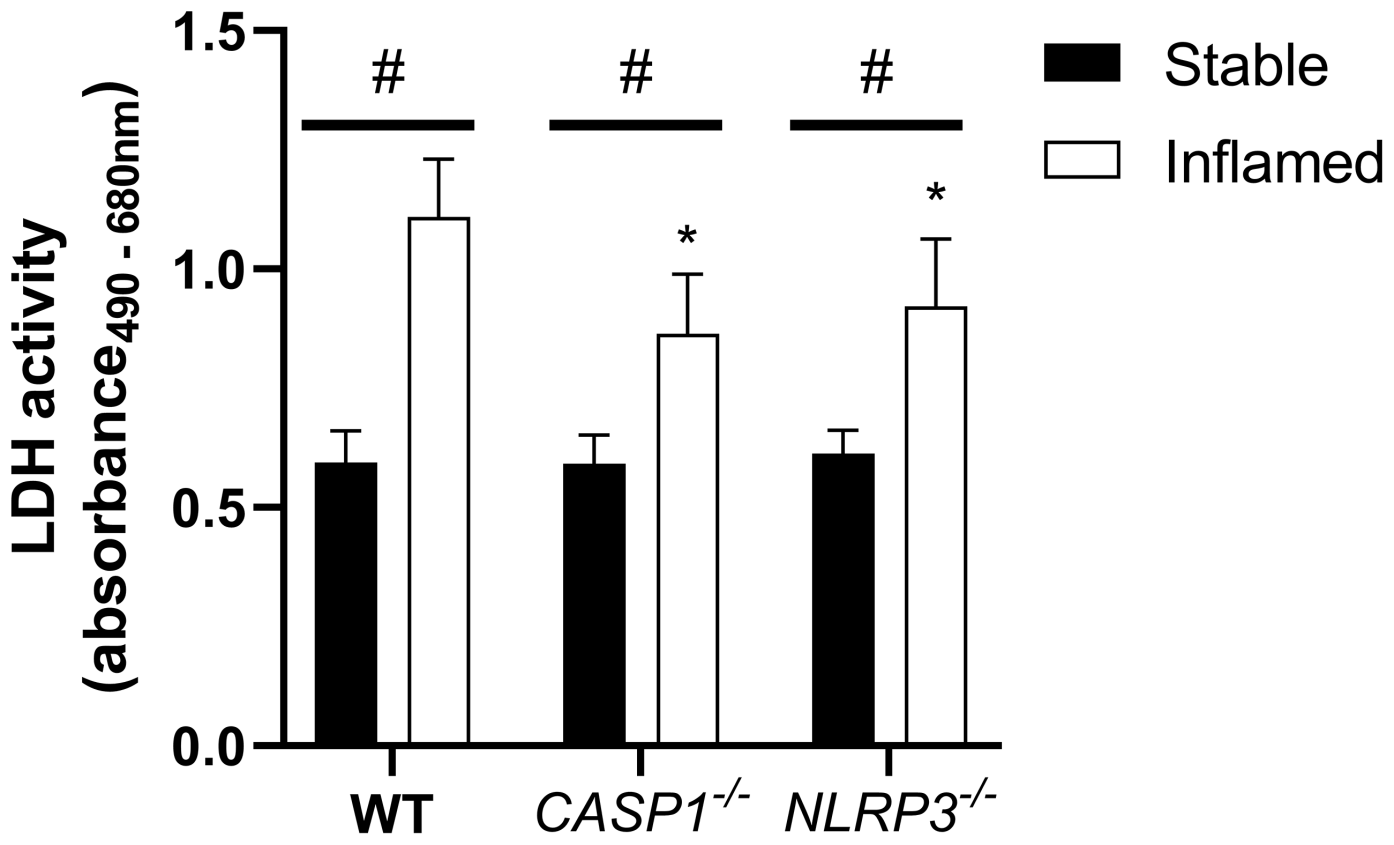
LDH activity in the apical compartment after 48 h of triple culture with WT, *CASP1^-/-^* or *NLRP3^-/-^* THP-1 cells. Mean + SD of N = 3. *p < 0.05 compared to the respective WT triple culture, ^#^p < 0.05 compared to the respective stable culture.

To assess inflammation-derived cytotoxicity in the epithelial layer, the LDH assay was applied after 48 h of triple culture (**Figure 3**).

In all three models, the LDH release into the apical supernatant in inflamed triple cultures was increased compared to the stable cultures. In both inflamed knockout triple cultures, LDH activity was significantly lower compared to the respective WT culture. No difference was observed between the stable cultures of the three models.

### Cytokine Release

**Figure 4:**
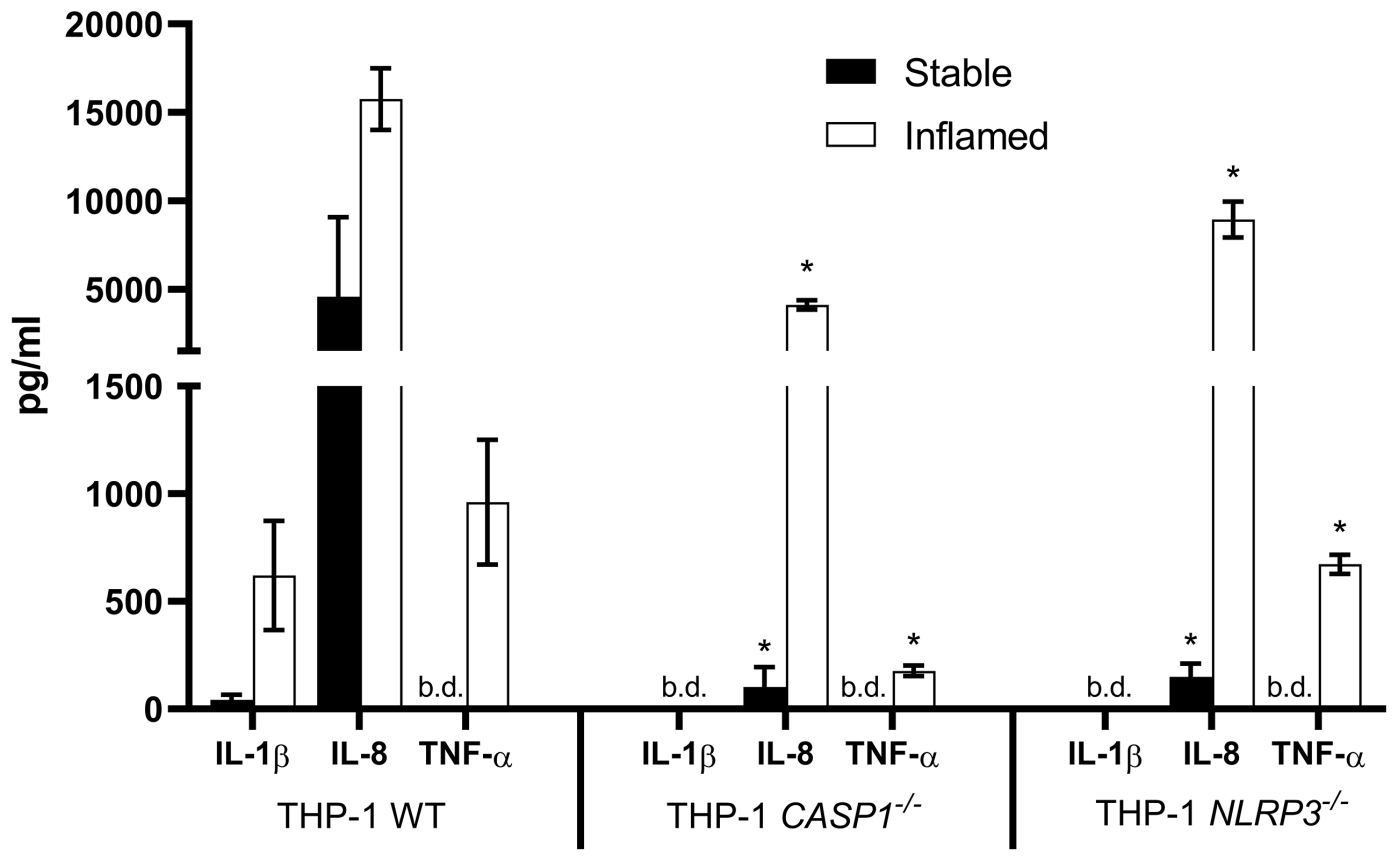
Cytokine release into the basolateral compartment after 48 h of stable (full bars) and inflamed (open bars) triple culture with WT, *CASP1^-/-^* or *NLRP3^-/-^* THP-1 cells, as assessed by ELISA. Mean ± SD of N = 3. *p < 0.05, compared to the respective WT triple culture. “b.d.” = below detection limit.

The cytokine concentrations in the basolateral supernatant of the three different triple cultures in stable or inflamed state were analyzed at t=48h (**Figure 4**).

As expected, no IL-1β was detected in the *CASP1^-/-^* and *NLRP3^-/-^* triple cultures, while its release was present in the stable WT culture and strongly increased in the inflamed WT culture. In all three triple cultures, the pro-inflammatory cytokines IL-8 and TNF-α were either strongly increased or only present in the inflamed state. IL-8 concentrations in stable or inflamed state were significantly lower in both knockout cultures when compared to the respective WT culture. TNF-α concentrations were below the detection limit in all three stable cultures. In inflamed state, TNF-α levels were significantly lower in the *CASP1^-/-^* and *NLRP3^-/-^* triple cultures compared to the WT.

### Gene Expression Analysis

**Figure 5:**
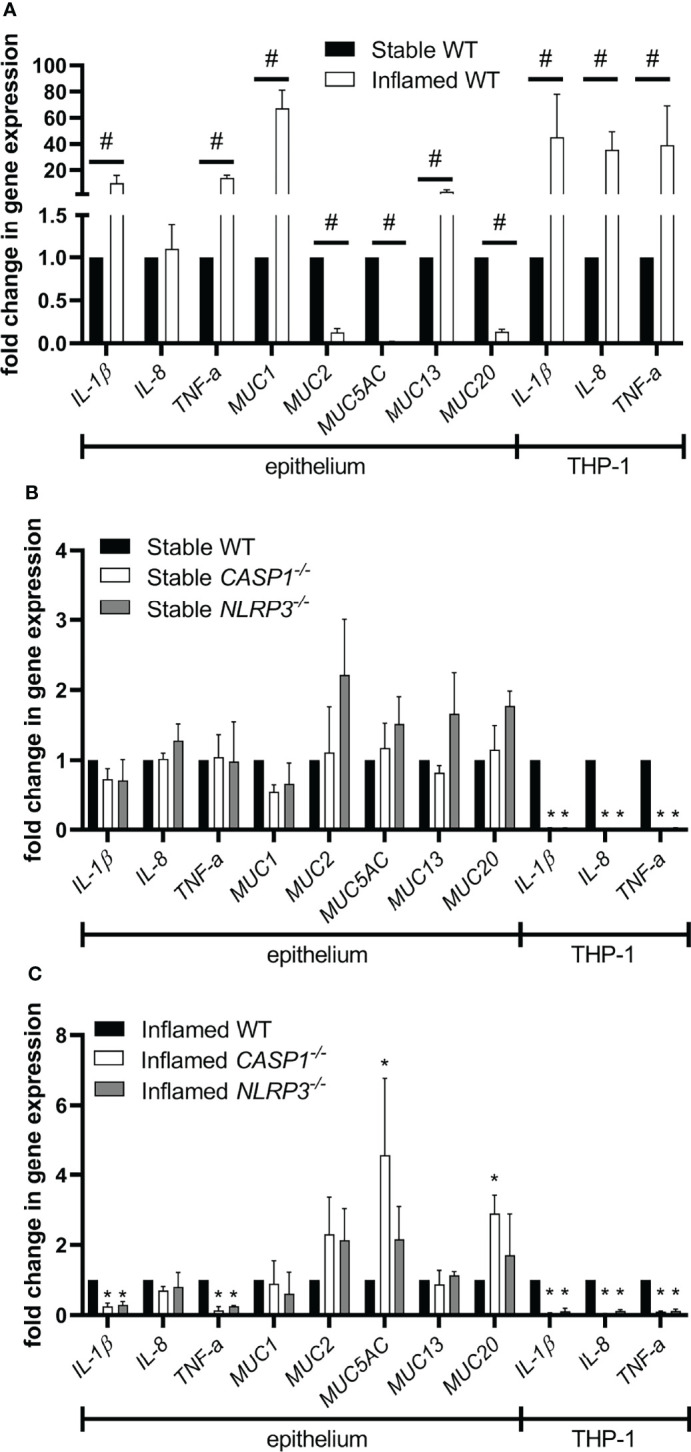
Gene expression in the epithelium or THP-1 cells after 48 h of triple culture. **(A)** Gene expression in the inflamed WT culture compared to the stable WT culture. **(B)** Gene expression in the stable knockout cultures compared to the stable WT culture. **(C)** Gene expression in the inflamed knockout cultures compared to the inflamed WT culture. Mean ± SD of N = 3. ^#^p < 0.05, compared to the stable control; *p < 0.05, compared to the WT control.

After 48 h of triple culture, gene expression of pro-inflammatory cytokines and mucins was analyzed in the epithelium and THP-1 cells (**Figure 5**).

Induction of the inflamed state in WT triple cultures resulted in a significant upregulation of *IL-1β*, *TNF-α*, *MUC1*, and *MUC13* in the epithelium (**Figure 5A**). Furthermore, *MUC2*, *MUC5AC*, and *MUC20* were significantly downregulated. In THP-1 cells, the gene expression of *IL-1β*, *IL-8*, and *TNF-α* was significantly increased in the inflamed state. When comparing the stable triple cultures, no differences in gene expression were observed in the epithelium (**Figure 5B**). At the same time, the expression of *IL-1β*, *IL-8*, and *TNF-α* was significantly lower in *CASP1^-/-^* and *NLRP3^-/-^* THP-1 cells in stable triple cultures. Comparing the inflamed triple cultures, the epithelial expression of *IL-1β* and *TNF-α* was significantly lower in both knockout cultures, while *MUC5AC* and *MUC20* were upregulated only in inflamed *CASP1^-/-^* triple cultures (**Figure 5C**). In both inflamed knockout cultures, the expression of *IL-1β*, *IL-8*, and *TNF-α* was significantly downregulated in THP-1 cells.

### MUC5AC Staining and Quantification

**Figure 6:**
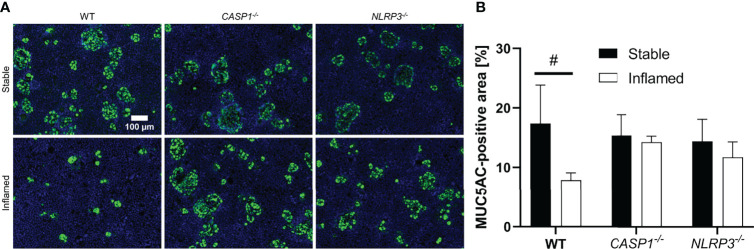
**(A)** Representative images of MUC5AC-stained epithelial layers of stable (top row) and inflamed (bottom row) triple cultures with WT, *CASP1^-/-^* or *NLRP3^-/-^* THP-1 cells. Blue: nuclei (Hoechst 33342), green: MUC5AC (Alexa Fluor 488). Images were acquired at 100x magnification. Scale bar (100 µm) applies to all images. **(B)** Quantitative analysis of MUC5AC-stained area in stable (full bars) or inflamed (open bars) triple cultures. Mean ± SD of N = 3, ^#^p < 0.05 compared to the respective stable culture.

To specifically investigate the presence and distribution pattern of mucus after 48 h of triple culture, the epithelial cell layer was stained for MUC5AC, a mucin produced and secreted by HT29-MTX-E12 cells (**Figure 6**).

All stable cultures showed large areas of up to several dozen cells, positive for MUC5AC staining distributed across the epithelial layer (**Figure 6A**), which accounts for ~15% of the total area. No statistically significant difference was observed between stable WT, *CASP1^-/-^* and *NLRP3^-/-^* cultures (**Figure 6B**). In the inflamed WT cultures, however, these areas are drastically reduced in size, and the area covered in MUC5AC is significantly reduced (~8%) compared to the stable WT cultures. Such difference between the stable and inflamed state was not observed in the *CASP1^-/-^* or *NLRP3^-/-^* triple cultures.

### Cytotoxic Effects of Triple Culture Supernatants on Epithelial Cell Monocultures

**Figure 7:**
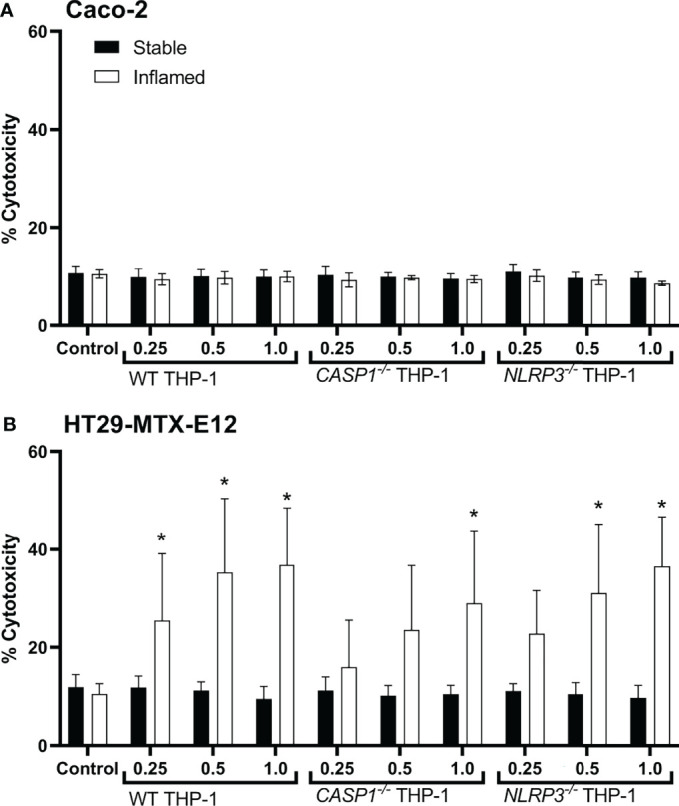
Cytotoxicity in Caco-2 **(A)** or HT29-MTX-E12 **(B)** monocultures after treatment with basolateral supernatants ( = 48h; relative concentration 0.25, 0.5 or 1) from stable (full bars) or inflamed (open bars) triple cultures (WT, *CASP1^-/-^* or *NLRP3^-/-^* THP-1 cells) for 48 (h) Cytotoxicity was assessed *via* LDH assay. Mean ± SD of N = 5, *p < 0.05 compared to the untreated control.

To investigate cell-specific, cytotoxic effects in triple cultures, monocultures of Caco-2 or HT29-MTX-E12 cells were treated with the basolateral supernatants of the triple cultures and the LDH assay was performed (**Figure 7**).

Treatment with triple culture supernatants did not cause any increased release of LDH in Caco-2 cells (**Figure 7A**). In HT29-MTX-E12 monocultures, however, the supernatants of all inflamed triple cultures resulted in cytotoxicity in a dose-dependent manner (**Figure 7B**). Treatment with WT supernatants caused a significant increase in cytotoxicity at a relative concentration as low as 0.25, treatment with *NLRP3^-/-^* supernatant at a concentration as low as 0.5, and treatment with *CASP1^-/-^* at a concentration of 1 (undiluted supernatant). No effects were observed in HT29-MTX-E12 monocultures when treated with the supernatants of stable triple cultures.

### Inflammation-Related Cytotoxicity and Gene Expression of Pro-inflammatory Cytokines in Intestinal Tissue Explants From WT and *Nlrp3^-/-^* Mice

**Figure 8:**
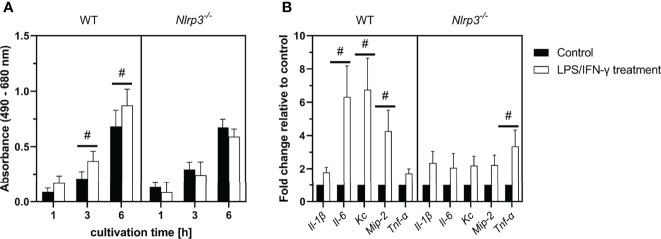
**(A)** LDH activity in the supernatants of LPS/IFN-γ-treated ileal tissue explants from WT or *Nlrp3^-/-^* mice after 1, 3, or 6 (h) **(B)** Gene expression in ileal tissue explants after 6 h of treatment. Mean ± SEM of N = 4, ^#^p < 0.05 compared to the untreated control of the corresponding time point.

To further investigate the role of NLRP3 in intestinal inflammation and compare the *in vitro* results to *ex vivo* data, we analyzed the role of the NLRP3 inflammasome on inflammation-related cytotoxicity and gene expression of pro-inflammatory cytokines in murine intestinal tissue explants. Ileal tissue from WT and *Nlrp3^-/-^* mice was incubated with the same pro-inflammatory stimuli as the triple cultures (LPS and IFN-γ) and cytotoxicity was assessed after 1, 3, and 6 h, while gene expression was analyzed after 6 h (**Figure 8**).

During cultivation, LDH activity in the supernatant of all explant cultures increased over time. No difference was observed between control tissue of WT or *Nlrp3^-/-^* mice. However, treatment with LPS and IFN-γ induced a significant increase of LDH activity after 3 and 6 h in the WT explants compared to the untreated control. This effect was not observed in the *Nlrp3^-/-^* explants.

In intestinal explants from WT mice, gene expression of the pro-inflammatory mediators *Il-6*, *Kc* and *Mip-2* was significantly increased after treatment with LPS and IFN-γ. An upregulation of these genes was not observed in treated explants from *Nlrp3^-/-^* mice. However, the expression of *Tnf-α* was significantly increased in the *Nlrp3^-/-^*, but not the WT explants after treatment.

## Discussion

The precise role of the NLRP3 inflammasome in intestinal inflammation is not understood yet. Although there is consensus about the crucial involvement of NLRP3 in intestinal homeostasis and inflammation, it is not clear if NLRP3 itself is a harmful or protective player (5). *In vivo* studies investigating DSS-induced colitis in *Nlrp3^-/-^* mice reported contrasting results on the severity of inflammation-related parameters compared to WT mice (12).

To investigate the role of NLRP3 in a different model of intestinal inflammation that does not involve animal experiments, we used an advanced *in vitro* human triple cell line culture of the healthy and inflamed intestine. Regarding inflammatory parameters, the inflamed version of the triple culture offers similar hallmarks as IBD, including an impaired barrier (34), inflammation-derived cytotoxicity (35), and increased concentrations of pro-inflammatory cytokines (36). IBD-associated loss of goblet cells has also been reported in rectal biopsies (37). We were able to replicate and detect all these effects when comparing the inflamed model with the stable, healthy model with WT THP-1 cells. Furthermore, we observed very specific changes in the mucin gene expression profile of the inflamed triple culture. *MUC1* and *MUC13*, both transmembrane mucins, were strongly upregulated during inflammation *in vitro*. Both mucins have been shown to be upregulated in IBD patients (19, 21). The membrane-associated *MUC20* was significantly downregulated during inflammation, which is in line with observations in IBD patients (23). *MUC2* and *MUC5AC*, secreted mucins, were downregulated during inflammation *in vitro*. A reduced expression of *muc2* has also been reported in colitis mice (38), while in contrast, an increased expression of *muc5ac* was observed in murine colitis (39). Apart from this discrepancy, the mucin expression profile of the inflamed triple culture model correlates very well with data from IBD patients and *in vivo* studies.

Investigating the effects of knocking out *CASP1* or *NLRP3* in the THP-1 cells, we found that both deficiencies significantly attenuated the aforementioned inflammatory parameters. Regarding barrier impairment, cytokine concentrations, and cytotoxicity towards goblet cells, the *CASP1* knockout tended to show some slightly stronger attenuating effect than *NLRP3*. When comparing the mucin gene expression profiles of inflamed triple cultures, the knockout of *CASP1* in THP-1 caused a significant upregulation of *MUC5AC* and *MUC20*. MUC5AC is predominantly secreted by HT29-MTX-E12 cells (27), which appear to be highly sensitive towards the contents of the basolateral supernatant, based on the cytotoxicity assessment in monocultures. This is in line with the MUC5AC staining of the epithelium, where MUC5AC-positive area is strongly reduced in the inflamed WT cultures. Taking these results together, it appears that HT29-MTX-E12 cells are proportionally more vulnerable during the inflamed state, compared to Caco-2 cells, which might explain the strong downregulation of *MUC5AC*. This downregulation is attenuated especially in *CASP1^-/-^* triple cultures, where the supernatant is less toxic towards HT29-MTX-E12 cells and more MUC5AC-positive cells remain on the filter after 48 h.

The expression and secretion of IL-1β, IL-8, and TNF-α, all crucial cytokines in inflammatory responses, is significantly reduced in LPS/IFN-γ treated *CASP1^-/-^* and *NLRP3^-/-^* THP-1 monocultures, as well as in inflamed *CASP1^-/-^* and *NLRP3^-/-^* triple cultures. This indicates that their release in THP-1 cells is directly dependent on (IL-1β) or influenced (IL-8 and TNF-α) by the NLRP3/CASP1 pathway. Furthermore, the gene expression of IL-1β and TNF-α is significantly lower in the epithelium of inflamed knockout cultures as well, meaning that the NLRP3/CASP1 pathway of the macrophage-like THP-1 cells affects the gene expression of the same cytokines in epithelial cells. While IL-1β is no longer detectable in knockout cultures, the concentrations of the cytokines IL-8 and TNF-α follow a gradient showing the highest concentrations in the WT cultures, followed by the *NLRP3^-/-^* and *CASP1^-/-^* cultures. Pro-apoptotic properties have been demonstrated for both IL-1β and TNF-α (40–42), while for IL-8, anti-apoptotic properties have been reported (43–45). Since IL-1β is completely absent in the knockout cultures, TNF-α might be the main cytokine responsible for apoptotic processes in the epithelium, which are still present, although to a lesser degree than in the WT cultures.

Our results show an enhanced susceptibility of goblet-like HT29-MTX-E12 cells towards inflammation-mediated cytotoxicity, compared to enterocyte-like Caco-2 cells. Strong synergistic cytotoxicity of TNF-α and IFN-γ towards HT29 cells has been described previously (46, 47). For our investigations, a high dose of IFN-γ is used to initially activate the THP-1 cells, so it is unlikely that the concentration of this cytokine differs significantly between the three inflamed cultures. However, analysis *via* ELISA revealed significant differences in TNF-α concentrations, which correlates to the cytotoxicity levels in inflamed triple cultures discussed above and HT29-MTX-E12 monocultures. Furthermore, TNF-α reportedly affects epithelial tight junctions and permeability (48). This might explain the lesser reduction of TEER in inflamed knockout cultures compared to the WT cultures. The relevance of TNF-α in intestinal inflammatory processes is well known, as anti-TNF-therapy is approved for the treatment of Crohn’s disease since 1998 (49). However, the interplay between the different cytokines, as well as other factors, is likely responsible for the differences in cytotoxicity and barrier impairment. Thus, further studies are necessary to investigate the exact role of TNF-α in this specific approach.

Summarizing the results of the triple culture experiments, the NLRP3/CASP1 pathway seems to play an adverse role in acute intestinal inflammation, as several inflammatory parameters are less severe in knockout cultures. The concentration of TNF-α, a secondary pro-inflammatory cytokine, is NLRP3-dependent and could be responsible for the alteration of specific cytotoxic processes during inflammation. The findings of the intestinal explant experiments support the harmful role of NLRP3: In contrast to tissues from WT mice, ileal tissues from *Nlrp3^-/-^* mice showed no signs of increased cytotoxicity after treatment with the same pro-inflammatory stimuli as the *in vitro* cultures. Furthermore, the gene expression of *Il-6* and the murine *IL-8* homologues *Kc* and *Mip-2* was increased after treatment only in the WT tissue explants. Surprisingly, the expression of *Tnf-α* was significantly increased in treated *Nlrp3^-/-^* explants, but not WT explants. As *Nlrp3^-/-^* mice also show a constitutively increased expression of *Tnf-α* in ileal tissue when directly compared to WT mice (see **Supplementary Figure 2**), we hypothesize that the upregulation of *Tnf-α* acts as a compensation for the lack of Nlrp3 as a defence mechanism in the knockout mice.

Interestingly, different studies investigating the role of the NLRP3/CASP1 pathway in mice with DSS-induced colitis are either in line with our findings or report the complete opposite. Bauer et al. (14) and Elinav et al. (50) also describe a harmful role of NLRP3 based on data from *Nlrp3^-/-^* mice, while Allen et al. (16) and Hirota et al. (17) report a protective role. Siegmund et al. (51) observed complete protection against DSS-induced colitis in *CASP1^-/-^* mice, while Dupaul-Chicoine et al. (52) reported that *CASP1^-/-^* mice died rapidly after DSS administration. Factors responsible for these contradicting findings are currently discussed and could include differences in microbiome and genetic background of the mice, as well as different protocols to induce experimental colitis (5). The triple culture, however, exhibits several differences compared to mouse models, including a drastically reduced number of different cell types and the lack of a microbiome. The method of induction of the inflammation and the duration of it differ as well. One major difference is the knockout of *NLRP3* only in macrophage-representing THP-1 cells, while in mouse models, all cell types are deficient of *Nlrp3^-/-^*. This means the mentioned *in vivo* studies investigated the role of NLRP3 during intestinal inflammation in general, while the presented triple culture experiments investigated the precise role of NLRP3 in macrophages. Zaki et al. (53) generated chimeric mice with *Nlrp3*-deficient hematopoietic cells (including macrophages) by transplanting bone marrow from *Nlrp3^-/-^* into WT mice, which represent a better *in vivo* analogue to our triple cultures with THP-1 knockouts. These mice were less susceptible to DSS-induced colitis than non-chimeric *Nlrp3^-/-^* mice, comparable to WT mice. The authors concluded that NLRP3 in non-hematopoietic cells (i.e. the epithelium) is more important for protection against intestinal inflammation than in hematopoietic cells. This is supported by the findings of Song-Zhao et al. (54); the authors showed that epithelial Nlrp3 offers early protection against bacterial-driven intestinal inflammation. Therefore, the NLRP3 inflammasome could have different, or even opposite roles during intestinal inflammation, depending on the cell type.

One limitation of the present study is the limited comparability to the *in vivo* studies. Inflammation itself is an *in vivo* phenomenon and only parts of it can be recreated *in vitro*. Furthermore, IBD is a chronic disease, while this *in vitro* model only allows investigations of initial, acute inflammatory responses. Nevertheless, our model exhibits several important hallmarks of the disease and compares well to data from intestinal tissue explants. Most importantly, it allows easy investigation and/or manipulation of separate cell types, which would be much more complex in an *in vivo* setup.

In conclusion, the use of THP-1 knockout cell lines in a triple culture model of the inflamed intestine presents a valid approach to investigate the role of the NLRP3/CASP1 pathway during acute intestinal inflammation. Our *in vitro* experiments suggest an adverse and pro-inflammatory role of NLRP3 in macrophages. The adverse role of NLRP3 was supported by data obtained from murine intestinal explants. The death of goblet cells and the subsequent changes in mucin expression and secretion appear to play an important role in acute intestinal inflammation, which seems to be indirectly linked to the activation of NLRP3.

## Data Availability Statement

The data presented in the study are deposited in the Mendeley Data repository, accession number 10.17632/5hy2sr37mm.2.

## Ethics Statement

The animal study was reviewed and approved by Landesamt für Natur, Umwelt und Verbraucherschutz (LANUV, Northrhine Westphalia, Germany, reference number 81-02.05.50.17.018).

## Author Contributions

MB: Conceptualization, Investigation, Analysis, Visualization, Writing. HR: Methodology. TW: Methodology, Investigation. AR: Methodology. RS: Conceptualization, Supervision, Writing. All authors contributed to the article and approved the submitted version.

## Funding

This project was funded by the Juergen Manchot Foundation, Duesseldorf, Germany through a Ph.D. scholarship for Mathias Busch.

## Conflict of Interest

The authors declare that the research was conducted in the absence of any commercial or financial relationships that could be construed as a potential conflict of interest.

## Publisher’s Note

All claims expressed in this article are solely those of the authors and do not necessarily represent those of their affiliated organizations, or those of the publisher, the editors and the reviewers. Any product that may be evaluated in this article, or claim that may be made by its manufacturer, is not guaranteed or endorsed by the publisher.
