# Supplementary material for: Investigating the Role of the NLRP3 Inflammasome Pathway in Acute Intestinal Inflammation: Use of THP-1 Knockout Cell Lines in an Advanced Triple Culture Model
Source: Front Immunol. 2022 Jul 13;13:898039. doi: 10.3389/fimmu.2022.898039 (PMC9326178; doi:10.3389/fimmu.2022.898039)
Supplement: Supplementary file 1 [file DataSheet_1.docx]

Supplementary Material

Supplementary Table 1 Human primer sequences and concentrations used for qPCR

| Gene |  | 5’ -> 3’ sequence | Concentration [nM] |
| --- | --- | --- | --- |
| *β-actin* | Forward primer | CCTGGCACCCAGCACAAT | 60 |
|  | Reverse primer | GCCGATCCACACGGAGTACT | 60 |
| *IL-1β* | Forward primer | GCCAGTGAAATGATGGCTTATT | 50 |
|  | Reverse primer | AGGAGCACTTCATCTGTTTAGG | 50 |
| *IL-8* | Forward primer | ACTCCAAACCTTTCCACCC | 60 |
|  | Reverse primer | CCCTCTTCAAAAACTTCTCCAC | 60 |
| *TNF-α* | Forward primer | ACTTTGGAGTGATCGGCC | 200 |
|  | Reverse primer | GCTTGAGGGTTTGCTACAAC | 200 |
| *MUC1* | Forward primer | AGACGTCAGCGTGAGTGATG | 37.5 |
|  | Reverse primer | GACAGCCAAGGCAATGAGAT | 37.5 |
| *MUC2* | Forward primer | GTCCGTCTCCAACATCACCT | 60 |
|  | Reverse primer | GCTGGCTGGTTTTCTCCTCT | 60 |
| *MUC5AC* | Forward primer | CAGCACAACCCCTGTTTCAAA | 60 |
|  | Reverse primer | GCGCACAGAGGATGACAGT | 37.5 |
| *MUC13* | Forward primer | CAGAGACAGCCAGATGCAAA | 60 |
|  | Reverse primer | CGGAGGCCAGATCTTTACTG | 37.5 |
| *MUC20* | Forward primer | GTGCAGGTGAAAATGGAGGT | 60 |
|  | Reverse primer | ACGCAGTAAGGAGACCTGGA | 37.5 |

Supplementary Table 2 Murine primer sequences and concentrations used for qPCR

| Gene |  | 5’ -> 3’ sequence | Concentration [nM] |
| --- | --- | --- | --- |
| *β-actin* | Forward primer | CGTGAAAAGATGACCCAGATCA | 100 |
|  | Reverse primer | CACAGCCTGGATGGCTACGT | 375 |
| *Il-1β* | Forward primer | GCCAGTGAAATGATGGCTTATT | 100 |
|  | Reverse primer | GATCCACACTCTCCAGCTGCA | 100 |
| *Il-6* | Forward primer | TGATGCACTTGCAGAAAACA | 100 |
|  | Reverse primer | ACCAGAGGAAATTTTCAATAGGC | 375 |
| *Kc* | Forward primer | GCACCCAAACCGAAGTCATA | 37.5 |
|  | Reverse primer | TGGGGACACCTTTTAGCATC | 60 |
| *Mip-2* | Forward primer | AAGTTTGCCTTGACCCTGAA | 375 |
|  | Reverse primer | AGGCACATCAGGTACGATCC | 60 |
| *Tnf-α* | Forward primer | TATGGCTCAGGGTCCAACTC | 60 |
|  | Reverse primer | CTCCCTTTGCAGAACTCAGG | 100 |

**ImageJ macro used for the automated analysis of fluorescent MUC5AC images**

dir = getDirectory("…\MUC5AC_quantitative_analysis")

list = getFileList(dir);

dir2 = getDirectory("…\MUC5AC_quantitative_analysis_results")
//setBatchMode(true);

for (f=0; f<list.length; f++) {

path = dir+list[f];

if (!endsWith(path,"/")) open(path);

if (nImages>=1) {

setAutoThreshold("Default dark");

run("Analyze Particles...", "size=100-Infinity show=Masks clear include in_situ");

run("Maximum...", "radius=80");

run("Minimum...", "radius=70");

run("Measure");

t=getTitle();

s=lastIndexOf(t, '.');

t=substring(t, 0,s);

t=replace(t," ","_");

t2= t +' bin';

rename(t2);

saveAs("Tiff", dir2 + t2 + ".tif");

selectWindow("t");

run("Close");

}

}
**Supplementary Figure 1.** TEER during 48 h of stable (circles) or inflamed (squares) triple cultures with WT (A), *CASP1^-/-^* (B), or *NLRP3^-/-^* (C) THP-1 cells.

**Supplementary Figure 2.** Ileal gene expression of *Nlrp3^-/-^* mice compared to WT mice. Mean ± SEM of N=4, *p<0.05 compared to the WT.
